# Listening to Hospital Personnel’s Narratives during the COVID-19 Outbreak

**DOI:** 10.3390/ijerph17176413

**Published:** 2020-09-03

**Authors:** Shir Daphna-Tekoah, Talia Megadasi Brikman, Eric Scheier, Uri Balla

**Affiliations:** 1 Kaplan Medical Center, Rehovot 7610000, Israel; drtaliamb1@clalit.org.il (T.M.B.); Eric.scheier@gmail.com (E.S.); uriballa@gmail.com (U.B.); 2 Faculty of Social Work, Ashkelon Academic College, Ashkelon 78211, Israel; 3 Faculty of Medicine, The Hebrew University of Jerusalem, Jerusalem 91905, Israel

**Keywords:** COVID-19 pandemic, healthcare professionals, first responders, listening guide

## Abstract

Healthcare workers (HCWs) facing the COVID-19 pandemic are required to deal with unexpectedly traumatic situations, concern about contamination, and mounting patient deaths. As a means to address the changing needs of our hospital’s HCWs, we conducted a narrative analysis study in the early stages of the covid-19 outbreak. A focus group of medical experts, conducted as the initial step, recommended that a bottom-up research tool be used for exploring HCWs’ traumatic experiences and needs. We therefore conducted 450 semi-structured in-depth interviews with hospital personnel. The interviews were based on Maslow’s Pyramid of Needs model, and the narratives were analyzed by applying the Listening Guide methodology. The interviewees expressed a need for physical and psychological security in the battle against Covid-19, in addition to the need for attachment and meaning. Importantly, we also found that the interview itself may serve as a therapeutic tool. In light of our findings, we recommended changes in hospital practices, which were subsequently implemented. Further research on HCWs’ traumatic experiences and needs will provide evidence-based knowledge and may enable novel approaches in the battle against Covid-19. To conclude, the knowledge generated by listening to HCWs’ narratives may provide suitable support programs for professionals.

## 1. Introduction

### 1.1. The Setting—COVID-19 in Hospitals

The moment that I was informed that we had become a COVID-19 department, I was devastated. This coronavirus is so frightening, and I knew that I could die from it. I am a person who needs to be in control, and I had lost control, I was so frightened. This entire new situation was scary—a situation of life or death. Moreover, I was in it. At the level of the team, we did not know what to expect, personally and collectively, as a department. I did not know what was expected from me as a social worker and what were the guidelines; everything was new. We created everything from the beginning, and I was scared.

*Emma, a social worker in the hospital’s Corona Department*

Studies on outbreaks of infectious diseases reveal the profound and broad-spectrum psychological impacts that disease outbreaks can inflict on healthcare professionals [1,2]. Experience with the SARS and Ebola virus outbreaks suggests that healthcare professionals are subject to extremely high levels of stress and emotional turbulence [3,4,5]. In dealing with the SARS virus, which is similar in some respects to the 2019 novel coronavirus (SARS-CoV-2), healthcare professionals were troubled by intrusive thoughts and images associated with SARS and exhibited symptoms of PTSD and substantial psychological distress [6], if not mental illness [7]. In addition, healthcare professionals feared that they would fall ill from SARS but were equally or more worried about infecting family members and other people [8]. Nonetheless, prior experience with pandemics, disasters, and major traumatic events has indicated that enhanced support for healthcare professionals enabled them to remain efficient and focused during these stressful events [9]. 

The on-the-job stress and burnout of healthcare workers (HCWs) employed in hospitals [10,11,12] is one of the key themes in studies of the trauma of these professionals in the corpus of literature on the cost of caring [13]. This recurring motif is not surprising, since the hospital environment is challenging in that it frequently demands holistic treatment of patients that integrates psychological elements with physical treatment: In the course of their routine hospital work, HCWs are required to deal with patients and their families who have experienced traumatic events and life-threatening episodes [14]. 

It was against this background that hospital HCWs the world over first encountered the global COVID-19 pandemic in December 2019 [15]. Facing this global public health event situated HCWs in an unbearable situation, with them being required to function under extreme physical and psychological pressure on both the professional and personal levels [16]. In parallel, they were often required to make impossible decisions, including how to deal with limited equipment, how to balance their own physical and mental welfare needs with those of their patients, and how to bring into line their responsibility to their patients with their responsibility to their family and friends. In addition, their wish to provide optimal care for severely ill patients was often constrained by inadequate resources. Thus, in the COVID-19 pandemic, frontline HCWs had to work under particularly intense stress levels in unprecedentedly difficult situations [17]—situations that may indeed cause stress, moral injury, and physical and mental health problems [4,17].

The unfolding pandemic has been compared to war, as described in “‘The Art of War’ in the Era of Coronavirus Disease 2019 (COVID-19)” by Maxwell et al. [18], who claim that the image of war is often used in the field of infectious diseases. Indeed, from the beginning of the pandemic, HCWs in the hospital setting have been faced with caring for patients with an incredibly contagious and life-threatening disease about which nothing was known and for which there was no known lifesaving treatment—a situation similar to a war. They were—and still are—handling life-and-death situations while simultaneously putting their own lives at risk. This factor has contributed to a real sense of danger among hospital staff, who have found themselves in the forefront of defense against the pandemic [19]. In parallel, HCWs are being required to deal with emerging challenges [20]. They are often required to develop, in an extremely short time, novel concepts and new interventions for unpredictable situations. The need for HCWs to be proactive results from the fast-growing numbers of critically ill patients, the lack of treatment modalities, and shortages of critical medical resources and staff. Like the general population, the hospital staff is struggling with the emotional stressors imposed by the pandemic. However, they are also faced with additional stressors and a rapidly evolving work environment that differs significantly from their pre-COVID routines [21,22]. 

Marchand-Senécal et al. [23] point out that specialized, dedicated COVID-19 teams could quickly be overwhelmed as numbers of cases increase markedly. They also hold that longer shifts and increased work intensity may lead to HCW fatigue and lapses in the use of the correct techniques for handling personal protective equipment (PPE). They illustrate this conclusion by citing initial reports indicating that about 4% of Chinese HCWs caring for COVID-19 patients were infected, with 15% of those HCWs being classified as severe or critical cases. It is thus not surprising that a survey of 1257 frontline nurses, physicians, and other HCWs who treated COVID-19 patients in hospitals in China found that the participants carried a psychological burden, with symptoms related to depression, anxiety, and distress [1]. Earlier studies on epidemic outbreaks have indeed revealed that medical personnel, particularly first responders, including physicians, nurses, ambulance personnel, and other HCWs, become emotionally affected and traumatized and display heightened stress and higher levels of depression and anxiety [24,25]. These findings are to be expected, since anxiety and the fear of being infected are aggravated as the risk of exposure is elevated. This heightened anxiety is exacerbated even further by the fear of transmission of the infection to loved ones. The need to maintain a sense of balance between professional duty, altruism, and personal fear for oneself and others thus often causes a mental conflict for HCWs [1]. In the guidelines published on 19 March 2020 by the World Health Organization, it was declared that, by virtue of their caring for and close contact with COVID-19 patients, the people most at risk of acquiring the disease are HCWs and that protecting HCWs is of paramount importance. 

In light of the above, at the beginning of the COVID-19 outbreak in Israel, we arranged a meeting in our hospital of an ad-hoc group of experts from different disciplines in healthcare and with different types of expertise. The recommendations of this focus group and the results of the interviews that were constructed and conducted as an outcome of these recommendations are described below.

### 1.2. Theoretical Framework

The theory of how humans usually function in daily life, as embodied in the ideas of Abraham Maslow, appear to be remarkably relevant to massive crises, especially to the present global crisis resulting from the COVID-19 pandemic [26]. Maslow’s theory establishes a hierarchy of human needs [27] and, as such, provides a framework to describe the needs of hospital personnel [28,29]. Maslow’s theory divides human needs into five categories. The first category, forming the base of a pyramid of needs, comprises physiological needs, such as air, water, food, shelter, sleep, and clothing. For medical personnel, Hale and his colleges [28] extend this level to include the basic determinants of good physical and mental health and safety. The first category is followed, in order, by four more layers: safety needs, such as personal security, employment, resources, health, and property; love and belonging, which includes friendship, intimacy, family, and a sense of connection; esteem and respect, which includes self-esteem, status, recognition, strength, and freedom; and, finally, at the top of the pyramid, the desire to become the best that one can be, relates to personal growth [30,31]. Maslow described each level as a separate need that relies on the previous need. However, modern-day theorists have modified this conceptualization into an overall concept in which each need coexists with the others [28]. We used semi-structured interviews based on Maslow’s pyramid to survey HCWs in our hospital. We then analyzed the interviews by choosing, from the umbrella of narrative analysis, the Listening Guide methodology to analyze the recorded interviews and their transcripts [32,33]. This qualitative research methodology was developed as an alternative analysis to conventional coding schemes used to analyze qualitative data [33]. It differs from other means of analysis in that it places emphasis on the psychological complexities of people through attention to voice as a manifestation of the psyche. The Listening Guide in its attention to voice—and silence—thus provides a way of exploring the interplay of inner and outer worlds and of bringing the inner world out into the open [33,34,35]. By focusing on different voices, on the dynamics and interplay of these voices within the interview transcript, and on the socio-cultural setting of the research, the Guide establishes a contextual framework for understanding and/or interpreting the narratives of the interviewees and thereby facilitates psychological discovery. The details of methodology are described in the Materials and Methods section [36].

Although every analytical process has its advantages and drawbacks, we chose the Listening Guide, since its intent is to capture the layers of perception and experiences of trauma and stress [37,38] that might otherwise remain unnoticed, thereby broadening the understanding of traumatic situations. By applying this methodology, we aimed to expand knowledge—and to generate new knowledge—regarding the mechanisms and the strategies used by hospital workers to cope with the COVID-19 pandemic. Specifically, we sought to reveal the overt and covert voices [39] emerging from the experiences of front-line workers and to examine how these workers describe and experience their traumas in their struggle to treat patients with COVID-19.

## 2. Materials and Methods

As mentioned above, being aware of the necessity to address the needs of the hospital’s HCWs, we conducted a study aimed to expand the knowledge of these needs during the first phase of the covid-19 outbreak. Specifically, the goal of the study was to enable the contrapuntal voices emerging from the hospital staff’s experiences to be “heard” and thereby to provide recommendations as to how to meet their changing needs as the crisis unfolded. By paying close attention the narratives of the hospital staff, we were able to address an additional aim, namely, to initiate the establishment of a data-based foundation for both immediate and future interventions, thereby expanding knowledge regarding the psychological mechanisms and strategies that front-line personnel use to cope with exposure to traumatic situations.

The first step of the research comprised the establishment of a focus group of HCWs, all trauma experts, in the hospital. On 18 March 2020, the focus group met to exchange thoughts about the evolving crisis conditions in the hospital and to find the means to evaluate—and indeed alleviate—the situation. The focus group comprised an Emergency Department physician and five medical-social workers with specializations in mental health and trauma. The decision to constitute a focus group of experts as the opening stage for this research project derived from the perspective that the novel coronavirus poses an unmet challenge to medical treatment and hence challenges HCWs in many unknown ways. The focus group was charged with delineating the new situation in the hospital and with deciding on the exact research methodology and research tool(s) that could be applied in a research project and possibly later as adjunct practical tools for dealing with the evolving crisis. The final question put to the focus group was: What is the next step in the research project?” The participants pointed out the need to conduct bottom-up interviews at all levels and sectors in the hospital as a means of understanding the emerging needs of the hospital personnel [40]. The focus group suggested conducting a survey based on semi-structured in-depth interview based on Maslow’s Pyramid of Needs’ model. The survey and results are described below.

The interview protocol involved questions designed to capture the HCWs’ ways of describing their unique experiences. To this end, the interview protocol comprised a standard set of questions, beginning with a request to share with the interviewer thoughts on what it meant to be an HCW in a hospital at the time of the corona crisis. This opening question was followed by encouragement to share thoughts on personal needs. More specific questions were used to clarify the stories as the interviews proceeded, such as: What do you need—physically or anything else? What distresses you at work? What would make you feel safer? What helps you to feel better? What helps you to know that you are appreciated? What motivates you to get up every morning for work? In a year from now, what do you think will have changed in your family, at the hospital, within yourself? Do you have something that you think important to add to the body of knowledge specifically about coronavirus or about trauma situations in general? 

Four hundred and fifty semi-structured interviews were conducted in three waves: the first in the middle of March 2020, before recognition of COVID-19 as a global pandemic (163 staff members; 36.2% of the interviewees); the second, two weeks later (157; 34.9%); and the third in the middle of May 2020, at the end of Israel’s national lockdown (130; 28.9%). Interviews were conducted with personnel [87 (20.2%) men and 344 (79.8%) women] serving a variety of functions in the hospital: physicians, nurses, pharmacists, respiratory therapists, department supervisors, laboratory technicians, social workers, and administrative workers from various sectors and with different levels of seniority (average 16.6 years; median 15 years, range:0-50) The breakdown of sectors and departments is given in Table 1. The interviews each lasted approximately 20 min to 1 h and were conducted under conditions of assured confidentiality.

As we indicated above, the interview narratives (in audio form and as transcripts) were analyzed by applying Gilligan’s Listening Guide methodology [41], which is comprised of four stages, as follows [37,42].

1. In the first stage, “Listening to the Plot,” attention is paid to the whole story of the interviewee. The researchers’ goal in this stage is to analyze the story in its context, similar to the analysis of an unfolding plot of a novel. The researchers identify recurring images and words, key metaphors, and dominant themes. The Guide also requires that the researchers document their own reflexive emotional and intellectual responses, thoughts, and feelings, as a means to better recognizing how their responses to the interviewee might affect their understanding of the narrative and the subsequent analysis. This stage is similar to the analysis modes in several of the qualitative thematic methods described in the literature [43].
2. The second stage, “I Poems,” is unique to the Listening Guide method. The second-stage listening and transcript analysis follows the use of the first-person pronoun “I.” Within a passage in the transcript, scholars underline every use of “I” together with the attendant verb and any seemingly important accompanying words and then paste these “I Voice” phrases together to compose an “I Poem.” This composite traces how the interviewee views herself/himself and the most prominent themes that preoccupy her/him.
3. The third step, “Listening for Contrapuntal Voices,” concentrates on how the interviewee talks about her/his or relationships with others. In this phase, scholars identify the multiple aspects of the story being told, often in multiple voices, with each voice (e.g., “You Voice,” “The Voice of Trauma and Stress”; see below) being underlined in a different color. The transcript thus provides a visual way of examining how the different voices change in relation to one another.
4. In the fourth and final step, “Composing an Analysis,” an interpretation of the interviews is developed that synthesizes what has been learned during the entire process by assembling the evidence drawn from the different instances of listening as the basis for composing the analysis. A summary analysis is then constructed [42,43].

The reported study conformed to internationally accepted ethical guidelines and relevant professional ethical guidelines and was approved by the institutional review board (IRB) of Kaplan Medical Center, Rehovot, Israel. To assure confidentiality, each participant was identified by a pseudonym.

## 3. Results

### 3.1. The First Step: “Listening to the Plot”

The narratives of the healthcare professionals covered a description of their experiences, explaining in detail their engagement with corona patients and their families and their relationships with members of the hospital staff. Two main themes emerged from the analysis of listening to the plot, the first of which was *preparing for war* in that the participants compared the situation in the hospital to the experience of preparing for war. Sara, for example, said: “*In our country, we know what a war is, and in the healthcare system we know how to function in the hospital during times of war, but still, this is a new war, a war that we have never handled, an invisible enemy, and it is frightening all of us*.” In similar vein, Doron said: “*to be significant, to be at the front is important. Before it was the army that was at the front, now it is the turn of the healthcare system to be at the front.”*

The second theme to emerge was that of *security and insecurity*, which related to two main aspects of the situation—fear of contamination and uncertainties derived from the assignment of HCWs to new teams and departments whose purpose and essence were unfamiliar. The idea of working in new and unfamiliar teams distressed the personnel, and their narratives reflected their feelings of insecurity. The changing reality was reflected by Dikla a nurse: *“In the Internal Medicine Department, I have been working for the past 18 years with my team, physicians, nurses, secretary—we have a common language. I felt especially secure in those days. How I will be able to use, in an efficient way, a new situation and new staff? This is ridiculous*.” Similarly, Tania, a social worker, said: “*This will increase the feeling of insecurity…think that the entire situation is new and scary; so, what will I do without my friends who I have been working with for years?”* In particular, the need to be protected during shifts was pronounced. As Sara told us: “*In order to continue to come here, I need to feel that someone is taking care of me. I do not care who in charge of that in the hospital, but I need to feel safe; it is essential for me.”*

According to the Listening Guide protocol, at this stage of the analysis, the interviewer should document his/her own reflexive emotional responses. We found that listening to the interviewees and reading the transcripts induced emotional concern for and feelings of empathy with our colleagues who were struggling with unbearable situations and ethical dilemmas. We were full of tears when we heard about the burdens of caring for patients and the cost of that caring. The interviews and the transcript readings thus gave rise to a range of emotions, including sadness, anger, compassion, and frustration, but also to pride in the devoted teams and to an appreciation of their devotion.

### 3.2. The Second Step: The “I Poem”

The second phase of the Listening Guide involves composing the “I Poem,” which is a core feature of the approach that serves to identify the active self. The process of composing the “I Poem” [44] can best be understood by examining some examples. Let us start by examining the transcript of the interview with Julie, a nurse in the ICU: *“I cannot believe it… because of the workload… it is only because of the workload… I have to tell you that I haven’t eaten for whole days…I grab something. It is not that there isn’t any food, but we don’t have the time and the needs of the staff draw you and you can’t ignore them; you need to respond to each one. At other times its different, of course. Here you can’t say anything to them. It’s the mask; it creates wounds on their noses, so I brought them cream. This kind of mask or any other; so, I saw masks in the grocery store and I bought them pink surgical masks so they would feel joy. Every day I am bringing something to make them happy. All the time. Yes, the protective equipment is a problematic issue by itself… I understand since I am involved in that; it depends on the equipment that comes to Israel, but it is not always suitable… this equipment is insane.”* In her “I Voice,” Julie demonstrated her personal difficulties in the Corona ICU and her difficulties in taking care of herself, even with regard to basic needs, such as food. However, as a manager in the ICU, her “I Voice,” expressed her competency in taking care of her ICU staff, as reflected in her “I Poem”:
Julie’s “I Poem”I cannot believeI have to tellI haven’t eatenI grab something [to eat]I brought them creamI saw face masksI bought them masksI am taking out [something to make them happy]I understandI am involved

Michal, a nurse, said: “I love my job, and I love the feeling of contributing. People around me, outside the hospital, talk about us [the HCWs]. I am in the frontline. It is pleasant and heartwarming.” Michal’s “I Poem” demonstrates her need to feel meaningful. In her “I Voice,” she expressed her caring and empathy for patients and fellow team members. She also expressed strength and resilience.



Michal’s “I Poem”I love [my job]I love [the feeling of contributing]I am [in the frontline]



Dina said: “I believe this will continue …. I discovered the richness of family and personal life, which reinforced things that I knew about myself and my [hospital] family—we are sturdy and dedicated and we cope well. I am filled with appreciation for the Infection Management Department that created a safe environment.” Her “I Voice” emphasizes her hope and capacity to keep the hard work as a result of teamwork.



Dina’s “I Poem”I believeI discoveredI knew [about myself]I am filled [with appreciation]



One can see that, in general, the “I Poems” reflect a layer of self-confidence and feeling of empowerment. This voice stands in contrast to other voices in the narratives that describe difficulties, stressors, and chaos, as described below.

### 3.3. The Contrapuntal Voices of Healthcare Workers

Irrespective of the contrapuntal voices embodied in the narratives, all the HCWs who worked in the hospital’s Corona Department stated that *the daily routine in the Corona Department was more complicated than that in the rest of the hospital*. The corona team concurred that the reasons for the differences were both physical and emotional and that acknowledgment should be given to the unique characteristics of the department. Sharon, a nurse, summed up this opinion very succinctly as: “*Corona—it is not extra work, it is completely different work.*” Against the background of this commonly held perspective, the third stage of the Listening Guide analytic technique nonetheless enabled us, the interviewers, to identify multiple voices that revealed different aspects of HCWs’ experiences and needs, including their attitudes towards the coronavirus pandemic, the staff and the hospital, and their own needs. The Listening Guide analysis of the focus group and the interviews identified five different contrapuntal voices—Trauma and stress, Security, Knowledge, Attachment, and Meaningfulness—intertwined not only with one another but also with the “I Voice” and the “You Voice” (see below). The contrapuntal voices and examples of quotes from the transcripts that represent these voices are described below.

#### 3.3.1. The Voice of Trauma and Stress

The hospital workers’ narratives reflected their direct exposure to traumatic events and the pervading presence of death in the hospital, as particularly manifested in the agony of seeing people dying without their families beside them and in the procedures for preparing the deceased for burial by special, double wrapping of the dead body as a precaution against contagion. For example, in response to the interviewer’s question “but you are accustomed to the death of patients, what is the difference?” Golda, a nurse, shared her feelings about traumatic moments after a patient’s death:

A deceased is a deceased but the separation from the family is extremely difficult, the wrapping process is a different from what you normally do in the internal ward. In addition to the regular wrap we put them in a nylon wrap and that is horrifying. A really unpleasant sight. It is like you put your patients in a plastic bag and you close it with a zipper. And then you cover with another bag but from the opposite side. An unpleasant wrapping of a patient since it is supposed to be isolated.

Similarly, Marina, a senior physician in the ICU, shared her difficulties in dealing with professional uncertainty and the absence of definitive information in the medical literature:

Look, the coronavirus is something completely new. A whole new disease that we do not have a clue how to treat, how to behave with it … and the craziest thing [is] that no-one in the world has the knowledge how to treat this disease, no knowledge-based expertise, no medical literature. So, you are constantly calling your colleagues in the country and around the world. Then, you are planning how you will cope with your first coronavirus patient. And then you are planning your second patient and the third. The decisions [as the head of the ICU] are just on your shoulders. They said to me: you are crazy … you are crazy; what are you doing? But I had to listen to myself, my instincts, and I said I have to go with my feelings and intuition. The decision is all yours. And what is most crazy is that you do not know what will happen next. Now it [the patient’s condition] is fine and five minutes later the patient can die and there is no-one to consult with because no-one knows [anything] about COVID-19.

In discussing the traumatic nature of their work in the ICU and the Coronavirus Department, Marina and Golda used the personal pronoun “you” in the masculine form when they spoke about taking decisions about life-and-death issues. We note here that in Hebrew, this usage of “you” in the masculine form is a generic usage that does not refer to the gender of the user. According to the Listening Guide methodology, the use of the masculine “you” hints at Marina’s and Golda’s difficulties in connecting emotionally to their traumatic experience of treating corona patients in the ICU [12,45]. Harel-Shalev and Daphna-Tekoah [45] have defined this Voice as the “You Voice,” a voice that enabled the HCW’s to distance themselves from recurring exposure to traumatic and painful experiences. It might represent a symptom of dissociation from traumatic events, not as a dissociative disorder, but rather as a coping mechanism allowing them to keep functioning as professionals.

Experiences such as these during routine work in unfamiliar situations were balanced by feelings of competency and an ambition to fight and succeed in the mission to conquer the novel coronavirus.

#### 3.3.2. The Voices of Security and Knowledge

These two voices—Security and Knowledge—are presented together since they are intimately intertwined. At the beginning of the crisis, the HCWs expressed their need for security and safety, primarily physical safety, and their need for crucial information and knowledge as a means to help them to feel more secure. With the progression of the pandemic, the HCWs became less anxious about physical safety and medical protection, as the hospital management met these basic needs and as the HCWs acquired the knowledge about how to protect themselves against contracting the disease. However, they still expressed the fear that, in the future, there could be a lack of equipment. According to Maslow, the most fundamental human needs are physiological, namely, air, water, food, shelter, and sleep. For medical personnel, Hale and his colleges [28] extended this level to include the basic determinants of good physical and mental health and safety. 

Orr, a nurse in the Corona Department, shared the following thoughts with us:

At the beginning of the corona outbreak, there was a lack of food, protective gear, and clothes and shielding eyeglasses to protect ourselves. We had to shower between the shifts, and there was a shortage of showers in the hospital, and we had to fight for the basic needs to be protected, especially during the weekends. It was horrible. Everyone was terrified. There was a lack of food in the Corona Department. At the beginning, I did not have what to eat during the day. I felt broken and choked …. There were shifts that I did not eat for almost 12 h.

Similarly, Sara, a single mother who moved to the Corona Department and worked 12 h shifts, said:

I did not have a life except the work at the hospital these past few weeks. I did not have a private life at all. I did not meet my family. I am tired all the time, I just want to sleep like a human being, to eat, to be away from the hospital and from the Corona that is all over; these 12-h shifts killed me. I am a single mother and I have a daughter. My daughter was all by herself at our house. It is unbearable; she was all by herself for all those days of the corona, and I was here taking care of other people.

The fear of being infected and of infecting others inside and outside the hospital, especially family and friends, was expressed vividly by the HCWs, mainly those working in the Corona Department. Dorit, a nurse, said:

There was constant anxiety and fear that we would infect others; we [at the Corona Department] felt like lepers … and then the isolation from my family since I was so afraid that I would infect them. I was isolated like a leper. My children could not go out to play with other children because I was terrified that I would infect my children and that they would infect their friends with coronavirus. At the beginning of the coronavirus, my daughter was so stressed out from this crazy situation.

The interviewees tied knowledge to the feeling of security and protection. Dan shared his feelings with us: “*The Head of the Department is constantly updating us … I do not feel detached … I feel secure, knowing where I stand.”* In contrast, Avi, an administrator, shared with us that: “ … *a lack of communication and information about what is happening at the hospital at the general level and not at the sector level bothers me. I am worried*.” In answer to the question, what helps you feel better? Yoav responded: “*Uncertainty concerns me—assessments of the situation and updates by my immediate supervisor would help me.*” And Ruth stated: “*I feel like I’m in the dark and don’t know what’s going on.”*

#### 3.3.3. The Voice of Attachment

As could be predicted from Maslow’s hierarchy of needs, the dominant needs for security and knowledge were replaced by the needs for belonging, love, recognition, and respect from supervisors and the hospital management as the pandemic progressed. The HCWs stated that a sense of family in the departments and the departments and friendship within their teams provided the much-needed sense of support during the pandemic. Moreover, the HCWs stated that without friendship, comradery, and a sense of belonging to a larger family, they would not have been able to work under such difficult conditions. For example, Sisi, a secretary at the hospital, said:

We were all a big family helping each other. I felt so close to all my peers; working together in such a tough time was different from what I had known in the last 26 years that I have been working in the hospital. As a team, we have become closer to each other, and I have discovered additional angels in my team …. In our department there is a sense of “togetherness” and comradery. Professionally, there will be changes; there are thoughts about modifying procedures in light of the current pandemic …. Relating to each other, currently feeling that we are a united and cohesive group.

Tova, a nurse in the ICU, said:

This period is a mixture of emotions. The reality is that everything is so new and unfamiliar. Nevertheless, the staff are so devoted to each other and struggling to do their best to help each other and changing shifts due to the lack of nurses. Sometimes they asked about treatment and I did not have an adequate answer. How I will say it? This is the period that we are re-inventing the protocols and rules of treatment. I am telling them that I am so sorry but there are no guidelines yet.

In addition, the interviewees expressed a need for recognition—appreciation and reinforcement—from their direct supervisors. For example, Tomer said: “*A good word, a compliment, and a positive attitude made me feel valued and…reassured.*” Orna said: “*A kind word makes my day …. It is essential for me to get feedback on my work and to know that I am doing my job well.*”

Another narrative relating to attachment was the need for a managerial presence, manifested as “managing by walking around.” This practice is considered one of the most important ways to build good manners and performance in the workplace and emphasizes the importance of interpersonal contact, open appreciation, and recognition [46]. The HCWs did indeed voice their desire for appreciation in the form of the need to meet management representatives in the various departments. Alma, for example, said: “*The presence of management in all departments and during all shifts made the staff aware that there was someone with them*.” She added: “*Personal appreciation by the management increases motivation and reduces concern….I would like to see more direct communication with management… in my team, I feel appreciated. I don’t feel I’m getting feedback from management*.” Lili said that when management came into the Corona Department to visit the staff and the patients, they asked her personally how she felt, and this was what helped her to feel valued. The HCWs were in agreement in their approval of the actions taken by the hospital management, judging the management’s conduct during the crisis to be appropriate and effective. Opinions of the following type were expressed: *Management worked well during the crisis*; *I want to thank the management for the adaptions that were made by mobilization of staff and change of policies and for taking the time to listen*; and *in my opinion, the hospital and management are doing well*.

#### 3.3.4. The Voice of Meaningfulness

The importance of feeling meaningful was verbalized by Hanna: “*Patients with coronavirus helped me to feel valued and meaningful, [especially] the conversations with the patients and the phone conversations with their families out there in their homes, so worried about their loved ones. I was there for the patients and their families, and it allowed me to feel meaningful and to want to continue treating patients.*”

## 4. Discussion

The global COVID-19 pandemic has challenged scholars and practitioners to find the means to alleviate stress and to treat the trauma experienced by members of the healthcare professions. Our study was designed to examine the experiences of HCWs during the first weeks of the COVID-19 pandemic, which may be considered as a massive traumatic event. The HCWs in this study, like other medical professionals caring for COVID-19 patients [40], have found themselves in a battle on two fronts: as hospital-based professionals fighting for their patients’ lives, giving rise to their perspective of themselves as combatants fighting on the frontline of a war, and as family members fighting to protect their families from exposure to the virus and paying the price for fighting the “new war.” As mentioned in the Introduction and Results sections, the image of war has become a common motif in discussions about the COVID-19 pandemic. Medical experts have even suggested that military strategies to be applied to outbreak management and have highlighted the importance of prioritizing healthcare staff capacity, as is done in military scenarios [18]. By documenting knowledge about HCWs, we thus contribute to a scholarly assessment and understanding of various elements of the new war—that against COVID-19. By seeking a dialogue with HCWs and, particularly, by engaging hospital staff in a genuine dialogue that deepens our understanding of the new battlefield and the “new health combatants,” we are now in a position to raise questions about “conventional wisdom” in the health system and to expand the knowledge about understudied topics in the new war [12,46].

We believe that to produce a deeper understanding of the experiences of frontline workers, whoever they may be, we should listen to them attentively [47]. Thus, the Listening Guide methodology provides a tool that can capture subconscious expressions through investigation of voices that are not usually otherwise revealed. By implementing the Listening Guide method in this study, we were able to explore more deeply the ways in which the HCW’s represent themselves and others—the ways in which they tell their story of the situation. In addition, we suggest that this methodology be integrated into the methods utilized in the healthcare arena and should be further explored in additional healthcare contexts.

During the interviews, the HCWs emphasized the high level of emotional intensity associated with long hospital shifts, the constant fear of death and of exposure to new and unfamiliar traumatic events, and the constant feeling of insecurity. The HCWs indicated that to cope with these emotional facets of their working environment, they needed a secure base. Issues of security and insecurity were revealed in different ways at different stages of the evolving crisis: At the beginning of the interviews, a clear majority of the staff emphasized the need for physical protection and the need to fulfill basic requirements, such as adequate food, a place to rest between long shifts, protective equipment, and showers in the Corona Department for use after their shifts. Another level of insecurity was emphasized by the need for personal recognition from direct supervisors and from hospital management. We found, however, that these needs changed as the pandemic progressed. At the beginning of the pandemic, when work environments were subject to change and scheduling remained uncertain, basic needs and physical safety were emphasized. In particular, respondents noted a shortage of protective equipment. The rapidly changing situation and the lack of supplies at the beginning of the pandemic crisis increased feelings of insecurity and intensified the importance of basic needs. However, as the crisis evolved, the need for security at the physical level was supplanted by a basic craving for security at the psychological and spiritual levels: The respondents focused on interpersonal relationships with their peers and their supervisors and their need for appreciation from their colleagues within their Departments and beyond and from management. The focus thus transitioned from personal health and well-being to a sense of social belonging, a need for respect and appreciation, and even a sense of personal and professional self-fulfillment as predicted by Maslow’s theory of needs [27].

Horesh and Brown [48] have encouraged trauma researchers “in the age of COVID-19” to employ all methods of scientific practice, including unique study designs and creative collaborations between disciplines with the aim to deepen the understanding of the health implications of the global coronavirus crisis. In particular, they indicated the need to develop novel methods for empowering and supporting medical personnel, as was done in the current study. Our status as researchers in the field of trauma and health and our particular, and perhaps unique, insider/outsider status as hospital personnel may raise questions about our specific situation and positionalities with regard to this study [46]. In response to such questions, we note that fieldwork, by its very nature, situates researchers among the community that they are researching, either as active participants or as observers or as a combination of the two [49]. As “researchers from within” [48], who are also HCWs, we felt obliged to study the experiences of HCWs in this unpredictable crisis. We were surprised by the high volume and the intensity of the traumatic experiences reported by the hospital personnel. We did not anticipate that HCWs who are accustomed to treating patients in the healthcare system would experience such insecurity and vulnerability. Importantly in this regard, we found that the anonymous qualitative interview—being conducted by skilled social workers—also served as a therapeutic tool and as a proactive means of communication with staff about their needs. The interviews enabled the HCWs to express their vulnerability and to acquire a sense of visibility and value. Brown [49] claims that vulnerability is the source of resilience and that vulnerability allows us to feel the emotions that we really crave—the need for human connection and the ability to belong and to “be seen” is something that every human being wants and needs. Brown holds that for us to be seen, we need to let others see us in a vulnerable state. Thus, the study framework—by enabling HCWs to express their concerns about “not been seen by friends or management” during the battle against the coronavirus—instilled a sense of confidence in the personnel with regard to their ability to communicate their vulnerability, needs, and concerns, particularly the need for personal and psychological security [49]. Bowlby [50], in his influential book “A Secure Base,” expands on the need for psychological security when he states that a basic component of human nature is the need for intimate emotional bonds and attachment. By enabling the hospital personnel to give voice to their needs and their insecurities, the interview itself became an intimate and emotional tool and a route of communication that ultimately allowed management to tailor various interventions to the needs of the employees and to increase the feeling of security in an extremely nonsecure situation.

Following the analysis of the data, the following practical interventions were developed by the hospital’s Social Work Department:

1. Ad-hoc meetings aimed at strengthening and supporting staff in transition (in that their departments had changed location and/or function to corona-related locations/functions) were arranged. COVID-19-dedicated teams were approached immediately before or after transition, and a focused, short intervention was conducted with all available staff members.
2. Telephone support for teams put in isolation after exposure to the coronavirus was established. 140 calls were made to support employees who were in isolation following exposure to patients infected with coronavirus.
3. Targeted short interventions were initiated for HCWs experiencing anxiety symptoms, and various relaxation techniques, such as eye movement desensitization and reprocessing (EMDR) treatment, were offered for trauma treatment.
4. Basic information was made available to employees exposed to patients hospitalized for COVID-19. Using the current research results, we created a brochure, in question and answer format, designed to provide information on employee health and rights, workplace guidelines, and the procedure that should be followed after an unwitting exposure to a patient with COVID-19.
5. A 24/7 hotline was opened for consultations and questions concerning mental or emotional distress.

The rationale for these interventions is embodied in the ideas of Santarone, McKenney and Elkbuli [51] that “maintaining the mental resilience of frontline workers involves offering solutions that allow them to perform their duties.” The study showed that the interventions reinforced the concept of the hospital as a protective organization, learning from the knowledge and experience of its staff rather than making assumptions to define these needs. We note that the interventions were not the main intention of the research but evolved from the needs of the HCWs, as expressed in the interviews. The need to generate immediate solutions to an acute crisis informed our decision to conduct a qualitative narrative analysis study. Nonetheless, as a “side-benefit” we have accumulated rich data, which we are now analyzing in greater depth in a mixed-methods study.

## 5. Conclusions

In light of the findings of this study, we recommend that leaders in the health care system should identify hospital HCWs as first responders (similar to combatants in the traditional military environment). Listening to hospital staff and exposing the price of the battle against the novel coronavirus may provide knowledge about new and understudied topics. This knowledge generated by narrative research, such as the current study, may, in turn, assist in providing suitable support programs for professionals fighting the new war in the health battlefield. Moreover, this new knowledge may be invaluable information and contribute to organizational resilience and coping strategies. To conclude, in this era, health care management should exhibit leadership by listening to HCW’s needs and adapting suitable interventions aimed to meet those needs.

## Figures and Tables

**Table 1:** Distribution of the interviewees according to sector and Department.

Sector N = 433	Medicine 72 (16.6%)	Nursing 169 (39%)	Admin 41 (9.5%)	Paramed * 26 (6%)	Other * 125 (28.9%)	
Dept. N = 381	Corona 50 (13.1%)	Internal med 105 (27.6%)	Surgery 13 (3.4%)	OBGYN ** 54 (14.2%)	Pediatrics 34 (8.9%)	Other ** 125 (32.8%)

Note: Values in the table are number (%) of interviewees that answered the specific question. The numbers do not add up to 450, because, in some cases, interviewees refrained from answering. * Paramed includes social workers, dietitians, physiotherapists, etc. Other includes kitchen workers, pharmacists, security personnel, housekeeping workers, etc. ** Department—OBGYN—Obstetrics and Gynecology; other includes hospital kitchen, pharmacy, security, housekeeping, administration, data and computing, etc.
